# Ailanthus Glandulosa

**Published:** 1896-12

**Authors:** C. L. Olds

**Affiliations:** Philadelphia, Pa.


### AILANTHUS GLANDULOSA.

C. L. OLDS, M. D., H. M., PHILADELPHIA, PA.

The common name of Ailanthus is the "tree of heaven." It has the aspect of a great sumach. At the time of flowering it gives off a very disagreeable stench, and seems to poison the air about it. Where it has been placed in parks it makes June the most unhealthy month of the year. Canaries hung out near it die. It was originally brought from China, on account of its rapid growth and beautiful appearance. Insects are poisoned by it; but in France it is cultivated for the sake of a species of silk-worm.

It has been used by the old school for tape-worm. The Chinese use it for dysentery. The provings have not yet substantiated this use.

It was first proved by P. P. Wells, of Brooklyn. His notice was first called to it by a case of poisoning in his own family. One morning his daughter got up sick. She went to the breakfast table and could not eat, then went to her room and had a violent fit of vomiting. Soon there came on headache, heat and redness of the face, dilated pupils, great intolerance of light, vertigo on rising—as soon as she tried to rise from a reclining position she would fall back. The pulse was small and rapid. She was drowsy and yet restless and anxious. In about two hours from the time she was taken sick she was insensible, with a muttering delirium. She had an eruption in patches, especially on the face and head. The eruption was miliary in character, dark, purple, in dingy patches, and the skin between the patches was livid. The pulse became smaller and more rapid, the skin became cold and dry, and when it was pressed by the finger the color slowly returned. Wells thought it was a bad case of malignant scarlet fever, and had little hope of saving his child; but in three or four hours the symptoms began to disappear, and gradually the attack wore off. Wells was puzzled at first, but found that it was not scarlet fever, but a case of poisoning by Ailanthus. His daughter had stripped off the bark from the tree and written letters on it with a pin, moistening the pin repeatedly in her mouth. In this child, and other provers, there has been an eruption on the body like the eruption of Ailanthus every year at the time of blossoming of the tree in June.

In this case of poisoning you see what a violent action this remedy has, what a deep action it has, producing a state like that of zymosis.

Running through the remedy will be found manifestations like those of scarlet fever, diphtheria, typhoid fever. *Putridity* runs through the remedy. The discharges are putrid, offensive; there are putrid ulcers, offensive breath. The eruption comes out in patches, miliary, dark, purple. The skin between the patches of eruption is of a brownish or livid hue or mahogany color. On the surface, in different parts, petechiæ are thrown out, large blebs form, containing a claret-colored serum—bloody serum; also fine blisters in different places. The imprint of the finger remains long on the skin. We find sordes on the teeth. Ulcers come in the throat—putrid, smelling like bad meat—with offensive oozing from the mucous membranes. The tongue is black and oozes blood.

From the beginning of these cases there is delirium, with great anxiety and restlessness, thirst for cold water, dilated pupils. Later on there will be stupor, but still the restlessness may continue. Prostration comes early and suddenly. The mind seems to be in a dreamy state—as if the patient were walking in a dream. She sees rats running about the floor, imagines that rats or snakes are running about the legs, or that a snake is about the throat. There may be a desire to cry all the time, or total indifference. There is loss of memory—she cannot think what she should reply. The moment she tries to rise up she falls back again—there is always vertigo on rising. There is headache with great fullness, burning in the head, aggravated by sitting up, aggravated by light, aggravated in one prover from 3 to 4 A. M. Add to these symptoms the eruption, the offensive discharges from the nose and throat—copious, thin, bloody, excoriating—the livid, swollen throat, the tonsils covered with fœtid ulcers, the tongue purple, swollen, perhaps with edges cracked, the urine suppressed—and you have a case of scarlet fever such as Ailanthus corresponds to—an exceedingly malignant case.

There are some symptoms of this remedy like Bell., yet Bell. is an entirely different remedy—you might say they were opposites. Some prescribers give Bell. for every case of scarlet fever in which they find delirium, restlessness, flushing of the face, dilated pupils, fullness in the head. This looks like Bell., but Ailanthus has all this and something deeper. The state of Bell. is one of active congestion, while that of Ailanthus is one of torpor, prostration, zymosis. Bell. has a bright red rash; in Ailanthus the rash is in dark patches, and the skin livid.

Ailanthus may be indicated in scarlet fever where the rash has been suppressed and symptoms like those enumerated come out. The mental symptoms will be present. If there is a rash, it is in purple patches; if not, the skin is blue and cold.

When this remedy is indicated in diphtheria the membrane seems to ulcerate at once. Large ulcers form, very offensive. The whole throat is purple, sensitive, swollen, oozing a dark, bloody, acrid discharge. Blood oozes from other parts. From the nose oozes a bloody, acrid discharge. Great crusts form about the nose, ulcers about the nose and lips; great scabs form on the upper lip. Perhaps great blebs form, containing claret-colored serum. The patient is stupid, the urine suppressed. This is something like Arum-tri., especially in the excoriating discharge from the nose; yet in Arum-tri. there is not as much stupor, but more restlessness. There is not the rapid ulceration of the membrane that there is in Ailanthus.

In cerebro-spinal meningitis this remedy will sometimes come into play. A chill is followed in a few hours by an eruption. The body is cold, the skin livid, mahogany-colored, purple, mottled, with dark purple spots.

In typhoid fever and in other typhoid states, we might expect this remedy to be useful. The patient is prostrated, comes down suddenly, is unable to meditate, does not comprehend what is said. We find a dreamy state of the mind, muttering, delirium, stupor, a drowsy yet restless state. He sees rats running across the floor or up his leg, feels a snake crawling up his leg or about his throat, has a startled look, with dilated pupils, and vertigo on rising. The chest has a dusky, bluish, livid appearance; ulcers form in the mouth—deep, corroding, bleeding; the tongue is dry, brownish, black, swollen, cracked; the teeth are covered with sordes. The abdomen is tympanitic, the urine very scanty or suppressed. The stool is frequent, small, thin, watery, offensive—perhaps of bloody mucus, and is passed involuntarily with the urine.

With the above symptoms this remedy may be indicated in erysipelas, very malignant form; also in blood-poisoning and dissecting wounds; and perhaps in child-bed fever.

The remedy has prolapsus of the anus. Hemorrhoids look like bullets; worse at stool, worse while kneeling, with a sensation of fullness. This sensation of fullness runs throughout the remedy. Diarrhœa alternates with constipation. There is a sensation at the anus as of a worm crawling about, with tingling, creeping numbness in different parts; also burning of the palms and soles.

Ailanthus has been used in asthmatic affections when the chest feels as if strapped tight, hooped up, as if the air cells were stuck together. The lungs feel tired, constricted, full, sore; there is burning in the chest, and difficulty in getting a breath. There is deep cough, with aphonia preceding it—exhausting cough, with copious expectoration of deep-yellow pus like mucus.

Throughout the remedy we see the anxiety and restlessness, the offensiveness, the prostration, the fullness everywhere—in the head, chest, abdomen; the burning in mucous membrane, burning discharges, burning headache, burning in the chest, in the throat, in the bowels.

In the zymotic diseases to which Ailanthus is particularly applicable, it will rarely be indicated unless the characteristic skin symptoms above enumerated be present; they are peculiar, and together with the prostration, restlessness, stupor, putridity, make the remedy stand out by itself in these much dreaded diseases. Its use has thus far been most frequent in isolated cases—not having been frequent in epidemics.

